# Very late onset post-transplant diffuse large B cell lymphoma in a liver transplant recipient with hepatitis B: A case report

**DOI:** 10.1097/MD.0000000000013063

**Published:** 2018-11-02

**Authors:** Fan Yu, Yuehua Huang, Yanying Wang, Zhuo Yu, Xinquan Li, Jiahong Dong

**Affiliations:** a Department of Hematology and Oncology; b Department of Hepatobiliary and Pancreatic Surgery, Beijing Tsinghua Changgung Hospital, School of Clinical Medicine, Tsinghua University, Beijing, China.

**Keywords:** hepatitis B, liver transplantation, lymphoma

## Abstract

**Rationale:**

Post transplantation lymphoproliferative disorder (PTLD) is a rare but severe complication. Epstein-Barr virus (EBV) is considered an important pathogen for PTLD and EBV deoxyribonucleic acid (DNA) load is widely monitored to detect PTLD early. Hepatitis B virus (HBV) infection is rarely reported to be related with PTLD. We report a case of EBV negative (EBV^−^), HBV positive (HBV^+^) diffuse large B cell lymphoma in a patient 12 years after liver transplantation.

**Patient concerns and diagnosis:**

A 52-year-old man complained of worsening appetite, abdominal distension, and pruritus. Abdominal computed tomography (CT) detected a huge retroperitoneal mass and pathology of the fine needle biopsy established the diagnosis of diffuse large B cell lymphoma. Virology showed active hepatitis B viral duplication and EBV DNA was negative.

**Intervention:**

Treatment modalities for this patient included: reduction and subsequent cessation of immunosuppression; antiviral therapy for HBV with entecavir and adefovir; conventional chemotherapy consisting of cyclophosphamide, epirubicin, vindesine, and prednisone, followed by radiotherapy. He achieved complete remission (CR) and was kept on entecavir treatment afterwards.

**Outcomes:**

He has been in remission for 2 years.

**Lessons:**

HBV infection might have played some role in this very late onset EBV^−^ PTLD patient. Therefore, HBV serology and HBV load should be monitored during the follow-up of HBV surface antigen positive (HBsAg^+^) transplant recipients and life-long antiviral therapy is required.

## 1 Introduction

Post transplantation lymphoproliferative disorder (PTLD) is a rare but serious complication among liver transplantation recipients, the overall incidence rate is reported to be 1% to 4%.^[1–3]^ PTLD can occur within the first 2 years after transplantation (early onset PTLD), or as late as decades after the surgery (late onset PTLD).^[3,4]^ The current World Health Organization classification identified 4 basic histologic types of PTLD: early lesions, polymorphic PTLD, monomorphic PTLD, and Hodgkin lymphoma/Hodgkin-like PTLD.^[5]^ Epstein-Barr virus (EBV) infection is an important and established pathogen for PTLD, especially early-onset cases with intensive immunosuppression. Nevertheless, EBV negative (EBV^−^) disease is reportedly seen in about 48% of the total population.^[6]^ EBV^−^ PTLD, showing similar pathogenic mechanisms with EBV^−^ lymphomas in immunocompetent hosts, is considered a different entity compared with the EBV positive (EBV^+^) PTLD, with distinct characteristics, including monomorphic histology, longer latency, and high-risk features.^[6]^ Strategies for managing PTLD include reduction in immunosuppression (RIS), surgery, radiotherapy, chemotherapy, and rituximab, determined by histology, stage, disease location, and patient's performance status.

Other viruses, such as cytomegalovirus (CMV) and hepatitis C virus (HCV), may also have some impact on the occurrence of PTLD.^[7,8]^ While Hepatitis B virus (HBV) infection is epidemiologically associated with diffuse large B cell lymphoma (DLBCL), little notice has been paid for the association between HBV infection and PTLD.^[9]^ Considering the risk for HBV reactivation in immunocompromised hosts after transplantation, HBV serology and HBV load may, beside EBV load, also be indicative of the PTLD risk in transplant recipients, especially, of late-onset PTLD risk.^[10]^ Here we report a EBV^−^, HBV^+^ patient who stopped antiviral agents 2 years after liver transplantation and developed DLBCL 10 years later.

## 2 Case report

### 2.1 Patient information

A 52-year-old male patient complaining of worsening appetite, abdominal distension, and pruritus for 3 months visited the hepatobiliary and pancreatic surgery department. There were intermittent night sweats and significant weight loss during the past 3 months. He underwent liver transplantation for hepatitis B cirrhosis and hepatocellular carcinoma 12 years ago. For immunosuppression he was treated with tacrolimus and prednisone right after the surgery for 3 months and then tacrolimus 1 mg twice a day ever since. He also took entecavir 0.5 mg once a day for HBV infection but stopped that by himself after 2 years. During the last decade, he was on regular follow up at a local clinic with normal liver function and normal liver morphology by ultrasonography. On physical examination, he had a hard abdominal mass about 15 cm in diameter without tenderness. He was suspected of recurrent hepatocellular carcinoma.

### 2.2 Clinical findings and diagnosis

Laboratory test showed normal liver function, an elevated lactate dehydrogenase level of 459 U/L (normal range 120–246) and a high HBV deoxyribonucleic acid (DNA) load. EBV viral load was negative. Virology data were shown in Table 1. Serum tacrolimus level was 7.2 ng/mL.

**Table 1:**
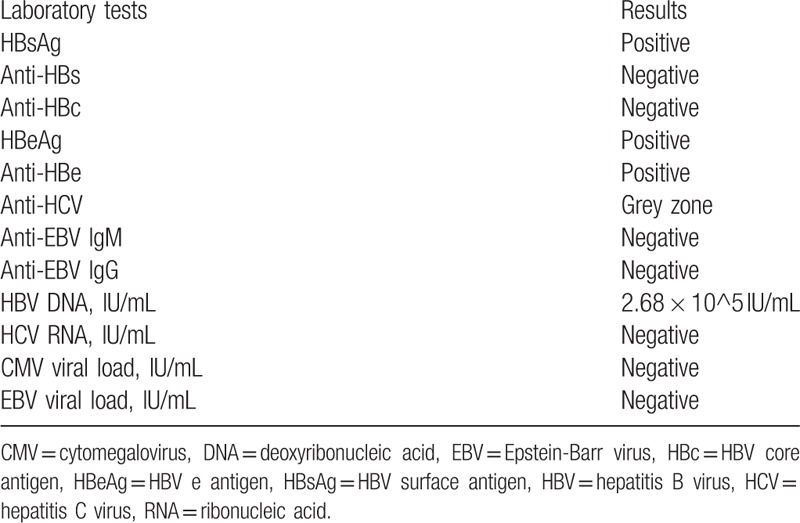
Immunological and virological tests results.

Abdominal contrast enhanced computed tomography (CT) revealed a retroperitoneal mass 127 mm × 114 mm × 119 mm in size, near pancreas extending to lumbar 4 vertebra, encompassing aorta abdominalis, right renal artery, inferior vena cava, and bilateral renal veins. There was mass effect on pancreas and kidney, resulting in displacement of the head of the pancreas and right hydronephrosis.

Biopsy of the mass was performed. Histopathology showed interspersed growth of the tumor cells in the rhabdomyus and immunohistochemistry showed cluster of differentiation (CD) 20(+), paired box-5 (PAX-5) (+), B-cell lymphoma (BCL)-2 (focal+), BCL-6 (+), CD10 (–), multiple myeloma oncogene (MUM)-1 (+), CyclinD-1 (–), Ki-67 (90%+), CD138 (–), CD3 (–), CD30 (–), anaplastic lymphoma kinase (ALK) (–), myeloperoxidase (MPO) (–). EBV-encoded ribonucleic acids (EBER) were negative by in situ hybridization. Monomorphic PTLD, diffuse large B-cell lymphoma was established.

Enhanced cervical and thoracic CT detected several small mediastinal lymph nodes, the largest 11 mm × 6 mm in size. Bone marrow biopsy didn’t reveal lymphoma involvement.

### 2.3 Treatment

Reduction of immune suppression was performed right after the diagnosis with close monitor of the liver function. With no sign of graft rejection, tacrolimus was tapered off. Antiviral therapy for HBV infection was initiated with entecavir and as the drop in HBV DNA viral load was not satisfactory, combination therapy of entecavir 50 mg and adefovir 10 mg once a day was administered. For lymphoma treatment conventional combined chemotherapy consisting of cyclophosphamide, epirubicin, vindesine, and prednisone, was given every 3 weeks. Rituximab was avoided because of the high HBV load. Per square meter of his body surface area, cyclophosphamide was given 750 mg on day 1 and epirubicin 80 mg on day 1. A maximum dose of 4 mg vindesine was given on day 1 and 100 mg prednisone per day was given orally for 5 consecutive days on days 1 to 5. After 6 cycles of chemotherapy, positron emission tomography–computed tomography showed residual mass 24 × 13 mm in size, with maximum standard uptake value 2.96. Therefore, he received consolidation radiotherapy for the involved field. Six weeks after radiotherapy, he was followed up with contrast enhanced computed tomography and complete remission (CR) was achieved according to the Lugano response criteria for non-Hodgkin's lymphoma.^[11]^

### 2.4 Outcome and follow-up

The patient was on continuous entecavir treatment for HBV infection and was followed up closely. His liver function is normal, HBV DNA has not been detectable and he is still in CR 2 years after radiotherapy.

## 3 Discussion

PTLD is a wide spectrum of heterogeneous diseases that commonly and severely complicate solid organ transplantation and allogeneic hematopoietic stem cell transplantation.^[3,12]^ Viral infections, along with immunosuppression, recipient age and ethnicity, and allograft type, are defined risk factors for PTLD.^[2,3,12,13,14]^

As far as viral infections are concerned, EBV primary infection or reactivation plays a key role in the pathogenesis. Under immunosuppression, T cell function is depressed and EBV infected and transformed B cells proliferate uncontrollably, which eventually lead to the development of PTLD. Nevertheless, about 1/3 of PTLD are EBV negative. Compared with EBV^+^ patients, EBV^−^ PTLD depicts different clinical characteristics with longer latency and monomorphic histology. Cytomegalovirus, hepatitis C virus, and Human Herpes Virus-8 are also reported in PTLD.^[7,8,15]^

The association between HBV infection and lymphoproliferative diseases was first suggested in 1970 and epidemiologically proved in recent years.^[16–18]^ The mechanism for HBV-related lymphomagenesis was also aggressively explored and genetic alterations identified may serve as therapy targets.^[19]^ HBV infection even aroused special attention in rituximab era because of the fatal hepatitis flare experienced by lymphoma patients getting anti-CD20 monoclonal antibody therapy.^[20]^ However, due to the limitation of patient number, there are very limited data on the relationship between HBV infection and PTLD, showing that in HBV reactivated patients after transplantation, the relative odds for developing PTLD were as high as 17.5.^[21]^ The risk for HBV reactivation in transplanted patients under immunosuppression is widely accepted, lifelong antiviral therapy in HBsAg^+^ cases has been required in most transplantation centers. Yet lifelong medication poses great challenges to patient compliance, as has happened in this case. Considering the prevalence of HBV infection, notably in Asian countries, and the increasing number of transplanted patients, lifelong anti-HBV treatment should be emphasized in patient education.

HBV related PTLD should be treated as an independent entity, given the necessity for antiviral treatment for HBV infection and the risk of hepatitis flare in patients getting Rituximab. As with other PTLD, reduction of immunosuppression is still a mainstay and should be conducted on a case by case basis to avoid graft loss. Combined chemotherapy and radiotherapy for DLBCL in this case led to CR, and appropriate treatment strategy should be based on histology.

This case is special in that the patient had high HBV viral load and developed PTLD 12 years after liver transplantation. Previous reports have put a lot attention on the relationship between EBV infection and PTLD and little attention on the relationship between HBV infection and PTLD. Accordingly, while EBVDNA viral load monitoring is a clinical routine during transplant recipients’ follow up, HBVDNA viral load monitoring is not uncommonly neglected. The lesson we can learn from this case is that HBV reactivation is related to PTLD and antiviral treatment should be kept life-long for HBsAg^+^ patients. Nevertheless, EBV^−^, HBV^+^, PTLD cases are rare and in this case direct evidence on the causal link between HBV reactivation and PTLD is lacking. Despite these limitations, it is reasonable to pay more attention to antiviral therapy and HBV status in HBsAg^+^ transplant recipients.

## Author contributions

**Conceptualization:** Fan Yu, Xinquan Li, Jiahong Dong.

**Formal analysis:** Yuehua Huang.

**Funding acquisition:** Zhuo Yu, Jiahong Dong.

**Investigation:** Fan Yu, Yanying Wang, Jiahong Dong.

**Methodology:** Yanying Wang, Zhuo Yu, Xinquan Li.

**Project administration:** Fan Yu, Jiahong Dong.

**Resources:** Yuehua Huang.

**Supervision:** Yanying Wang, Xinquan Li, Jiahong Dong.

**Validation:** Zhuo Yu.

**Writing – original draft:** Fan Yu.

**Writing – review & editing:** Yuehua Huang, Xinquan Li.

Fan Yu orcid: 0000-0001-8166-2468.
